# The Impact of the Content of the Label on the Buying Intention of a Wine Consumer

**DOI:** 10.3389/fpsyg.2018.02761

**Published:** 2019-01-14

**Authors:** Diana Escandon-Barbosa, Josep Rialp-Criado

**Affiliations:** ^1^Gestión de Organizaciones Department, Pontificia Universidad Javeriana Cali, Cali, Colombia; ^2^Economia i Empresa, Universitat Autònoma de Barcelona, Barcelona, Spain

**Keywords:** labels, purchase intention, expert consumers, novices, gender

## Abstract

This paper aims to analyze the influence of the content of the label of wine bottles on the purchase intention of wine. The sample was 114 individuals (51, 32% women and 48, 67% men) inside simulated Supermarket at Javeriana University of the city of Cali (Colombia). They were viewed for 2 min in front of a grocery shelving with 100 wines. The study uses eye tracking to estimate the behavior related to wine attributes included on the label of wine bottles, specifically the denomination of origin, nutritional information, and health warnings. The authors use the hierarchical model methodology, which generates a pattern of relationships among variables. Among the results, it is possible to determine a consistent model for purchase intention, where the mentioned components of the label that are related to wine attributes and their interactions constitute important factors in the possibility of influencing a purchase intention. However, when executing a group division of the audience attending to the experience in wine consumption, it is evident that based on their experience, consumers read the labels’ information differently, causing certain effects in their buying intention.

## Introduction

To study the consumer behavior and their process for decision making, the marketing field has increased by theories, methodologies obtained from different sciences such as psychology, sociology, economy and other (Stasi et al., 2018).

Analysis of consumer behavior validates that for the same type of product there are different consumption groups. As a consequence, consumer product producers as well as scholars have created different market segmentations, specific to each product, which allow for the development of deeper and more intensive analyses to understand each population group. Since 1980 there have been studies about the different wine consumption segments, organized by socio-demographic characteristics like lifestyle, geographical location, and others (Szolnoki and Hoffmann, 2013; Gluckman, 1990). Within the existing segmentations, this research focuses on two groups: by type of expertise in consumption and by gender.

Gender equality has evolved in all dimensions of society and its customs, having a direct influence on the consumption habits and the roles played by men and women within the family and among shopping and purchasing behaviors, given that today’s egalitarian values greatly affect purchasing decisions. A considerable number of studies have included gender as a key determinant in their research to explain the different perceptions of men and women (Buss, 1988; Baltas, 1997; Eagly and Steffen, 2000). However, few studies have demonstrated the role of gender as relevant to analyze the influence of label content on purchase intention (Slama and Williams, 1990; Miquel et al., 2017). This few research are demonstrative of gender is relevant for consumer behavior studies. In general, the effectiveness of content of labels is associate of have confidence information (Liao et al., 2015; Songa et al., 2019) and it generate emotional connection for decision-marking for purchase.

On the other hand, there are further types of consumers who are classified by their level of expertise or knowledge of the product. Expert consumers are those who know the product because they tend to be recurring consumers, while non-experts are those sporadic consumers who have low product information. In this sense, studies in the literature have shown that there are two types of highly differentiated consumers due to the volume of information they can process and understand when selecting and buying a product. Although it is recognized that they are two different market segments, there are few studies that explore these differences. In the case of the content of the label, no study that analyzes the differences between expert and non-expert consumers has been found.

This project aims to give continuity to the schemes that analyze the influence of the content of the product’s label on the purchase intention for the product (Sherman and Tuten, 2011; Tang et al., 2015; Danner et al., 2017). Additionally, this study focuses on analyzing the presence of the denomination of the product’s origin, nutritional information, and health warnings on the product’s label in relation to consumption, and it seeks to examine how these aspects relate to the purchase intention within the two classifications – expert and non-expert – and by gender. In this case, the selected product to analyze the effect of these label components on purchase intention is wine.

Nowadays, the main wine-consuming countries have seen this product changing from being sold only in specialized stores to being bought mostly through retail channels in mass-market stores (Lick et al., 2017). This implies that the process carried out by consumers for their selection and purchase is quite complex, as it is influenced by many factors and fulfills the conditions of the retail market, for example, that it is bought without being able to prove it (Lick et al., 2017). According to Lick et al. (2017), the three key objectives to achieving a product sale in the retail channel must be met by packaging. These are: attract the consumer, provide information, and persuade him/her to make the purchase.

The receptive interaction between the consumer and the product plays a significant role in the purchase intention. This is because nowadays the packaging is no longer a purely functional tool but has become a mechanism to establish a close relationship with the consumer. The packaging of wine, especially the main bottle label, is responsible not only for transmitting information about the product as such, but it also serves to connect with the emotions and expectations of the consumer (Krishna et al., 2017).

For consumers of this product, choosing a wine at the retail location is not an easy task given its wide variety. Therefore, the descriptions provided on the wine bottles’ labels are usually very useful information for the consumer at the moment of their choice. Sherman and Tuten (2011); Tang et al. (2015), and Danner et al. (2017) have pointed out that wine labels play a meaningful role in the consumer’s choice, influencing their expectations and emotional responses and generating changes in the acceptability of the product.

To cover our objectives, this article is divided into the following sections: a first part includes the theoretical framework and the approach to the study hypothesis; this is followed by the methodology used to test the hypothesis, the discussion of the results, and conclusions.

## Background Literature

With regard to consumer behavior, the purchase intention is a key aspect that can be defined as the probability of buying the labeled product (Hawley et al., 2013). According to Goh et al. (2016), the purchase intention is an effective tool to predict the buying process, given that consumers’ purchase decisions will be driven by their intentions. The use of the information presented on labels has an impact that has been highlighted in many different investigations (Campos et al., 2011; Thøgersen et al., 2017), which have reported different aspects to consider, for instance the preference for short labels on the front of the product in contrast to long descriptions on the back (Williams, 2005; Herpen and Trijp, 2011).

Accordingly, the denomination of origin is an influential factor in the purchase decision for wine, especially for those people who do not have prior knowledge and experience in their consumption (Campos et al., 2011).

Furthermore, expert consumers read the label’s information differently compared to non-expert consumers. According to Mitchell and Dacin (1996) and Carsana and Jolibert (2015), while non-expert consumers try to make a quick choice and detail few wine attributes, the denomination of origin is one of the few attributes used by this group of consumers. In contrast, the expert consumer has a longer purchase process, in which he/she considers different sources of additional information from the denomination of origin.

Therefore, the denomination of origin is a characteristic used mainly by non-expert consumers, and it may even be irrelevant for expert consumers (Mueller and Szolnoki, 2010). In the case of non-expert consumers, the denomination of origin allows them to obtain sufficient information to increase their purchase intention given that it allows them to gain generic information about the quality of the wine just by knowing the place of origin (Read et al., 2016).

Yet another part of the label that acts on the intention of purchasing wine by expert consumers and, to a lesser extent, by non-expert consumers is the nutritional information. Its influence has been shown to be greater in women than in men. Several investigations about the effectiveness of the use of nutritional information on labels and its influence on consumer decisions have been carried out (Drichoutis et al., 2005; Annunziata et al., 2016). It should be noted that the labeling requirements for alcoholic beverages differ depending on the country and have a limited scope with regard to the information on labels of other products (Mueller et al., 2010).

Apart from the obligatory nature of this type of information on the labels, the effectiveness of the information resides in the consumer’s interest in it and in his or her capacity for understanding it (Mueller et al., 2010). In the case of wine, though labels are often neglected with respect to nutritional information, it has been demonstrated that for experienced consumers it is a key source of information because it provides them details about the quality of the wine (Kleef and Dagevos, 2015) and allows them to attend to decisions about the authenticity of the product (Youn and Kim, 2017).

Nevertheless, in the case of the influence of nutritional information by gender, no evidence of differentiation has been found in the literature. However, when women read more information on the product labels, this creates a greater contact with the nutritional information of the product. Additionally, compared to men, women may have a broader intention to buy based on the nutritional information because they base their decisions on other information available or on the opinion of others. In this sense, since wine is a product of social prestige, with high publicity about its nutritional and health benefits (Yap and Tan, 2017), it can generate a larger effect in this population group.

Health warnings on the labels of alcoholic beverages have been established in some countries, according to regulatory authorities, to make consumers aware of the harmful effects of alcohol on the human body. These warnings provide information like alcohol levels, restrictions for specific population groups (minors, pregnant women, and people who will drive after consumption), information about the presence of some potential allergens, and maximum daily values of alcohol consumption. Such warnings vary depending on the country.

The presence of warnings on the labels has been widely examined by producers, but some entities such as the World Health Organization are concerned about not having more effective measures. Kersbergen and Field (2017) have claimed that these warnings have limited effect, given that consumers do not pay enough attention to this part of the label. Thus, the scarce information related to health displayed on wine labels and other alcohol beverages is focused on promoting moderate consumption, which leads the consumer to consider the negative effects of the consumption of these products.

As a result, health warnings are visibly displayed on the label with bold letter sizes or colors to discourage consumers from all population groups (experts, non-experts, men, women, etc.). Nowadays, however, a large number of mainstream publications have upheld the positive effects of wine on the health of the people who consume it, offsetting these cautions.

Consequently, a model that examines how the components of the label can affect the purchase intention for wine has been developed. From previous studies in the literature, different hypotheses have been proposed according to the type of consumer (wine experts and non-experts) and by gender to find possible similarities or differences in these population groups.

### Hypotheses

The wine label must be able to display the information in the most practical and precise way so that the consumer can make his/her purchase decision, in lieu of an alternative product. The purchase of wine in the retail channel takes no more than 10 min (Schnettler and Rivera, 2003); therefore, both the information on the label and the valuation of the product will be closely related to the amount of time consumers take to observe and analyze it (Krishna et al., 2017).

The interaction of different components of the wine labels – such as the denomination of origin, health warnings, and nutritional information – and their influence on the purchase intention has not been analyzed in previous studies. This interaction can be highly relevant in experienced consumers because they seek the most information available to be certain about their purchase.

In the case of considering the denomination of origin and the nutritional information, though no previous works have analyzed this combination, it is important to emphasize that these components are the ones that occupy the most visual space within the wine label. In this sense, in the case of people who observe the label, this information can help to generate a classification of the product or some stimulus (Gutman, 1982).

With regard to expert consumers, they use the denomination of origin only in combination with other characteristics, or when the other information is incomplete or ambiguous (Perrouty et al., 2006). In addition, these consumers significantly value the greater amount of information available on the label, given that they can distinguish and separate the different types of wines into categories by analyzing the exhibited information (Solomon, 1997).

In general terms, the information on the label such as the denomination of origin, nutritional information, and health warnings are key aspects to transfer to the consumer the experience that can be produced when enjoying the product. For expert consumers, it is highly relevant to have the largest amount of information available to make a selection. Expert consumers make a deeper and more detailed analysis of the label than do non-expert consumers. What’s more, the level of understanding of the information is also different between the two types of consumers. For instance, for experts, having more information generates a positive impression because it manages to implement different categories of analysis to make the purchase decision. Yet a greater amount of information requires further cognitive processes and may exceed the comprehension capacity, especially for non-expert consumers, creating a sense of confusion (Beverland, 2006). Consequently, we postulate:

**Hypothesis 1:** The thoughtfulness in health warnings strengthens the positive influence of the attention in denomination of origin and purchase intention, but this relationship strengthens as nutritional information of both increases, for expert and non-expert wine consumers.

On the other hand, the examination of multiple components of the label from the gender perspective is associated with differences in information processing between men and women (Rodgers and Harris, 2003). For example, in the case of buying wine, women seek information that is quick and easy to process, such as the designation of origin and nutritional information, to make their decision, while men tend to make a more detailed analysis of that information.

A recent study demonstrates a highly accepted point of view which analyzes the gender differences in terms of self-construal (Cross and Madson, 1997). This vision intends to analyze the differences from the same mental process, which includes aspects such as the processing of information, emotional differences, and the willingness to explain the human behavior associated with their decision making (Maddux and Brewer, 2005). Then, these natural differences are united with social conceptions to create self-construal in a different way for each of the genders.

Research on brand and consumer behavior found that exist differences between male and female. Female usually attention more strongly on the text, but male focus more on images (Putrevu, 2004; Goodrich-Hunsaker et al., 2011; Aschemann-Witzel et al., 2018). This is because males and females use different selectivity models to process information.

Females use more comprehensive model with a selection form than those utilized by males (Goodrich, 2014). Additionally, female can read more careful that males when reading information (Leong and Hawamdeh, 1999), allowing that female present a significantly greater tendency to read or explore product information than males because they wish to know more aspects of product content (Shaouf et al., 2016; Aschemann-Witzel et al., 2018).

Consequently, men and women differ in the sort of information they assume as relevant and on which they consider making their judgments and finally making the purchase decision (Wolin et al., 2002). Women are linked with being more visual and using different sources of information to be able to interpret the product signals and to give a more informed opinion (Meyers-Levy and Sternthal, 1991), while men tend to be more frequently engaged in intuitive processes with an interpretation of images (Hass, 1979; Joo et al., 2018).

In the case of women, to a greater extent than in men it is evident that during the wine selection process there is a high predisposition toward reading and observing the content of the label (Atkin et al., 2007). Some studies carried out in multiple regions of the world have argued that women tend to carefully consult the bottle label and its information when they are not sure about which wine to buy (Atkin and Sutanonpaiboon, 2007). To sum up, we put forward:

**Hypothesis 2:** The consideration in health warnings strengthens the positive impact of the attention in denomination of origin and purchase intention, but this relationship strengthens as nutritional information increases in women compared to men consumers of wine.

**FIGURE 1:**
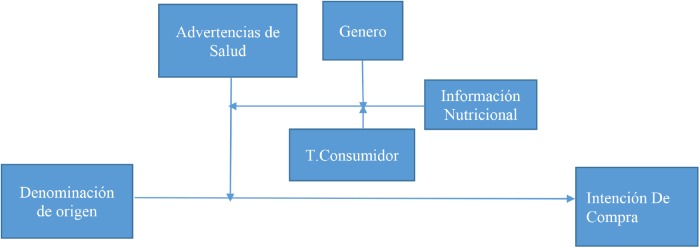
Model.

Accordingly, the theoretical model proposed for this research is visualized in Figure 1.

## Materials and Methods

### Sample

The selected participants for the study were men and women who are part of Javeriana University in Cali, Valle del Cauca, Colombia. The university has a laboratory that simulates a supermarket in an area of 125 m^2^. This laboratory was equipped with more than 100 wines. The sample (114 individuals, 51.32% women and 48.67% men; with a high concentration (45%) in ages between 25 and 54 years old) was related to the Javeriana University of the city of Cali (Colombia) and it was a good representation of the Colombian population attending gender and age standards (50.8% women and 49.2% men – 41.82% between 25 and 54 years old).

The study was conducted through eye tracking as an investigation of the impact of visual images because it is principal form to acquire information (Songa et al., 2019) on tastes and preferences through the wine attributes: denomination of origin, nutritional information, and health warnings.

This study was carried out in accordance with the recommendations of World Medical Asociation’s Declaration of Helsinki, it was performed in concordance with Editorial and Ethics Committee of the FCEA and Institutional Committee, Javeriana University. The protocol was approved by the Editorial and Ethics Committee of the FCEA and Institutional Committee. All subjects gave written informed consent in accordance with the Declaration of Helsinki.

To qualify, participants must have had a smartphone with internet access and have reported consuming at least one glass of wine per week. Participants did not enroll in the study until they completed a pre-intervention evaluation period that was used to confirm whether they met the minimum wine consumption required for inclusion (8 oz per week or at least 7 drinks per week). On average, the randomly selected participants reported consuming 23 oz of wine during the period prior to the intervention. This process in which participants must report their behavior in real time and context is called Momentary Ecological Evaluation (Stone and Shiffman, 1994; Gibbons, 2017).

During the pre-registration period, participants had to answer at least 14 of the 28 messages sent by EMA (90.5%) to be selected for the study. Participants who missed more than two messages were given the opportunity to repeat the entire process with adequate compliance before being withdrawn from the study. Only three people managed to be admitted on their second attempt.

This procedure was repeated for the 114 participants who were placed under laboratory conditions of the Universidad Javeriana Cali with an interval between trials of 1500 ms. Participants were observed for 2 min in front of a grocery shelving unit with approximately 100 wines, where it was possible to show more than 1200 observations regarding the participants and the combinations of the information on the wine label.

### Measurements

Participants in this study were classified as expert and non-expert depending on the frequency of their wine consumption and the number of years of consumption.

In general, non-expert is characterized by low years of consumption and low weekly frequency; while expert is characterized by several years of consumption and high weekly frequency.

Moreover, to check Hypothesis 2, participants were divided by gender as a dichotomous variable to segment the wine consumers; women were assigned the value of 0 and men the value of 1.

To measure purchase intention, the eye-tracking technique used these indicators:

– Dwell Time: Total average time of visualization of the area, regardless of the number of fixations.
– First Fixation Duration: Time in milliseconds of the first fixation.
– Revisits: How many more times the person entered to see the area of interest.
– Fixation Count: Number of fixations.
– Average Fixation Duration: Average length for each fixation, in milliseconds.

With this information, a factor analysis was performed to obtain a common factor that explained 85% of the total variance of the original variables (KMO = 0.91); the common factor presented an eigenvalue of 3.5.

Additionally, we did a testing of wine for consumer who answered between two option: “I certainly would buy” Or “I am not sure would I buy” The purchase intention ratings increased when consumer affirm “I certainly would buy” but decrease when the answer that “I am not sure would I buy or not”. In General, purchase intention is high relation with actual purchase (Paired *t*-test: 1.0236 *p* > 0.1).

In contrast, for the measurements of the denomination of origin (DO), nutritional information (NI), and health warnings (H) variables, we wondered whether consumers would fix their gaze in these areas. Hence, these three variables were also dichotomous, with 0 if the consumer did not look at the area and 1 if the consumer observed it.

#### Robustness Checks

In our experiment, wine’s consumer tested and answered about their preferred wine and which wine they would buy in the future (Purchase real). We performed robustness checks on our results. For robustness test purposes, we present results based to find heterogeneity between purchase intention and purchase final. We find no significant heterogeneity “between-group” (Hotelling T2 = 13.51, *p* = 0.14) for wine consumer in Colombia allowing to conclude that purchase intention have a very relation with purchase final. Additionally, We utilized a jackknife resampling technique to check the robustness in our results. This technique is convenient for estimating the robustness of these types of models (Chambers and Chandra, 2013). The results confirm that sub-survey estimations (jackknife) are very comparable to the original results – providing assurance for the original estimations.

## Results

Initially, we estimated an ANOVA model in which the dependent variable was related to the purchase intention, and the independent variables were DO, NI, and H; subsequently we added double and triple interaction effects. To conclude, a graphic analysis of the interactions for each group of consumers was carried out. The variables of gender and type of consumer were also included to identify the differences between these groups.

In general, the principal effects did not have a significant impact, and only the consideration of the health warnings was relevant according to the types of consumer (expert, non-expert). Moreover, it was found that the double effects between the main variables were significant for the two study groups, except for the double effects that included gender or type of consumer. This result illustrates that wine consumers have to look for combined information to make a decision. In the case of triple effects, unique values were achieved only in the interactions among DO, H, and gender because the combination of DO and gender was significant for women and less relevant for men (*t* = 3.1: women = 0.710, *p* > 0.10, men = 1.13, *p* < 0.05).

**FIGURE 2:**
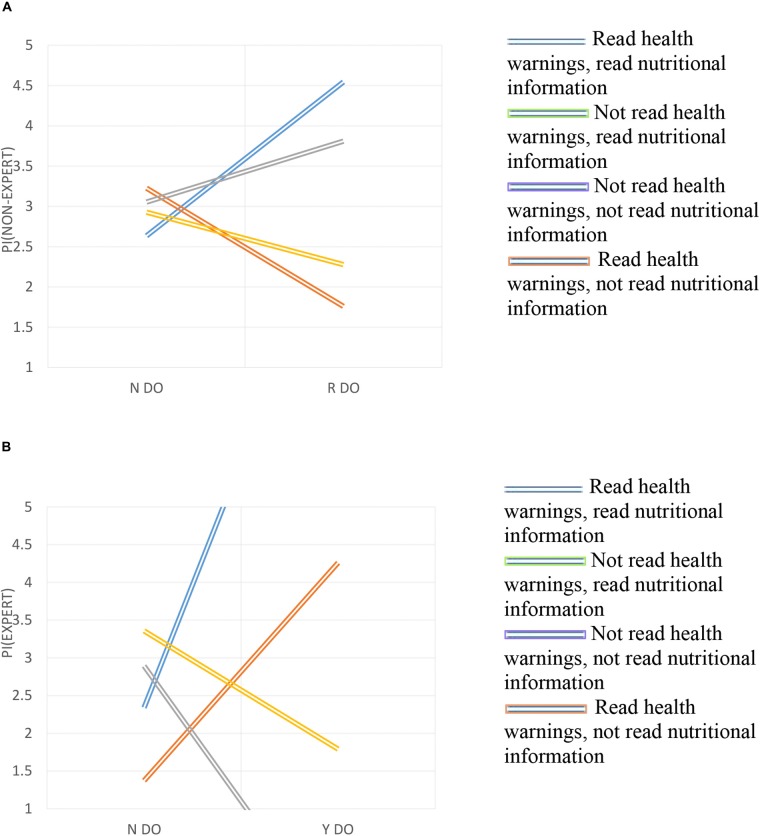
**(A)** Triple interaction in non-expert wine consumers. **(B)** Triple interaction in expert wine consumers.

**FIGURE 3:**
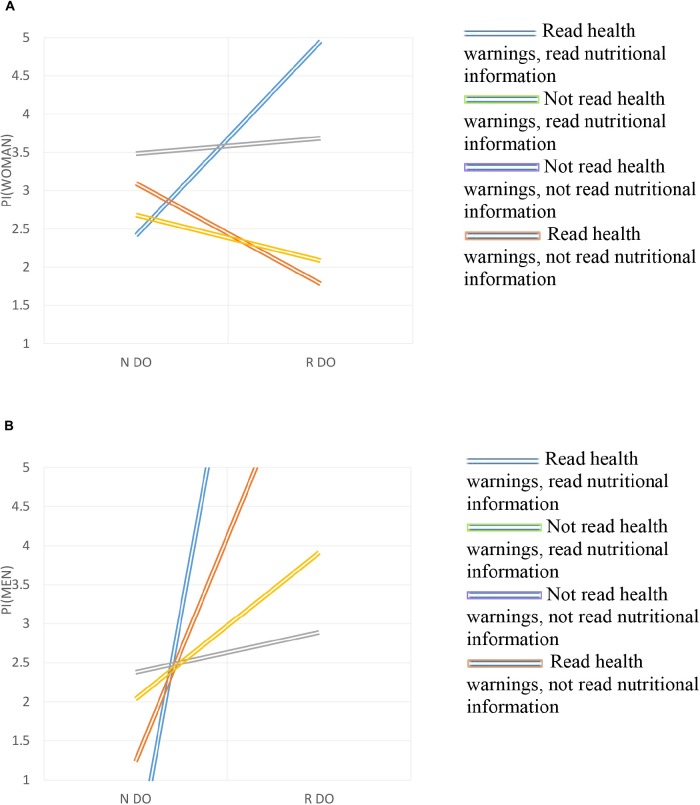
**(A)** Triple interaction in female wine consumers. **(B)** Triple interaction in male wine consumers.

Summing up, to check Hypotheses 1 and 2, which were the main areas of investigation, Figures 2, 3 show the magnitude and statistical significance of the coefficients reported in Table 1 (Dawson and Richter, 2006).

**Table 1:** ANOVA for each consumer group.

		Non-expert consumer		Expert consumer		Woman		Man	
Variable	Coef	B	*P*-value	*b*	*P*-value	*b*	*P*-value	*b*	*P*-value
DO	b1	0.023	0.77	0.507	0.30	1.29	0.08	0.081	0.59
NI	b2	0.001	0.93	1.505	0.08	1.57	0.05	0.050	0.67
H	b3	0.654	0.13	0.625	0.25	0.951	0.13	1.20	0.04
DO^∗^NI	b4	0.021	0.78	2.35	0.03	1.31	0.08	0.24	0.35
DO^∗^H	b5	0.587	0.10	0.38	0.37	0.710	0.19	1.13	0.05
NI^∗^H	b6	0.013	0.82	2.75	0.02	1.70	0.04	0.028	0.75
DO^∗^NI^∗^H	b7	0.318	0.02	1.82	0.05	1.38	0.07	0.988	0.07


The Y axis represents the obtained values for the purchase intention for each group (by gender and level of expertise). In the X axis the different values of the independent variables are presented: (i) the consumer does not read the denomination of origin (0); (ii) the consumer reads the denomination of origin (1); (iii) the consumer does not read the nutritional information (0); (iv) the consumer reads the nutritional information (1); (v) the consumer does not read health warnings (0); and (vi) the consumer reads health warnings (1). Two levels of the moderation variable are presented (Aiken and West, 1991; Jaccard et al., 2003).

In contrast, Figure 2 shows the interaction effect by type of consumer. In Figure 2A, if a non-expert wine consumer does not read the health warnings, the designation of origin positively affects the purchase intention only if the consumer also reads the nutrient information (Function 3) but decreases if the consumer does not read this information (Function 4). Then, a low effect on the purchase intention is achieved when the consumer brings into consideration the three components of the label (DO, NI, and H) because it is confuse for them.

Figure 2B shows that expert wine consumers who do not read the health warnings but do read the denomination of origin decrease their purchase intention to a greater extent if they do not read the nutritional facts (Function 3) rather than reading this information (Function 4). When men read the health warnings, it is likely that if they also read the denomination of origin they can increase their purchase intention more if they obtain additional information about nutrition (Function 1) than if not (Function 2). Therefore, the expert consumer will increase his or her purchase intention when obtaining information on the three components of the label.

This is consistent with Hypothesis 1, which affirms that the interaction of denomination of origin, health warnings, and nutritional information increases the purchase intention of both expert and non-expert wine consumers.

If the size of the effect is examined, it is found that for non-expert consumers the purchase intention is 4.54 and for experts it is 8.86. Therefore, Hypothesis 1 is verified, though there are significant differences among the groups (*p* < 0.01): experts have greater effects on their purchase intention by attending to the three components of the label than do non-experts.

In this Graph, we provide behavioral differences between expert and non-expert wine consumers, and it is possible to visualize how decision making of expert is different to non-expert: the expert consumer will increase his or her purchase intention when obtaining information on the three components of the label, but non-expert consumer won’t.

Figure 3A shows that if women do not read the health warnings, the reading of the denomination of origin is more likely *not* to be carried out to improve their purchase intention than when they read the nutritional information (Function 3), but decreases the purchase intention when they do not read the nutritional information (Function 4). When women read the health warnings, they are likely to read the denomination of origin to aim for increasing their purchase intention to a greater extent if they obtain additional information on the nutritional facts (Function 1), but decreasing their intention to purchase if they do not read the nutritional information (Function 2). In general, for women, the effect on purchase intention increases significantly when they look at the three components of the labels.

In the case of men (Figure 3B), if they do not read the health warnings, the reading of the denomination of origin is less likely not to be carried out, affecting their purchase intention to a greater extent if they only read the nutritional information (Function 3) rather than not reading this information (Function 4). When men read the health warnings, it is likely that if they read the denomination of origin it will increase their purchase intention to a greater extent if they obtain additional information about nutrition (Function 1) than if not (Function 2). In general, for men the interaction effect of the purchase intention is high when the three components of the label (DO, NI, and H) are considered.

This is not consistent with Hypothesis 2, which states that the interaction of DO, NI, and H more positively influences the purchase intention of women compared to men. If the size of the effect is evaluated, it is found that for women, the purchase intention is 4.05 and for men it is 12.88. Therefore, there are significant differences between men and women (*p* < 0.05); men show a greater effect on their purchase intention by considering the three components of the label.

There are significant differences between the time men take to make the decision and what women take (*t* = 4.87), being higher for women (mean of women = 145 s; mean of men = 64 s). This implies that women take decisions less rapid due to the time dedicated to look for and analyze prior information before taking a decision, while men make it in a much more agile way.

To sum up, the verifications of the hypothesis are shown in Table 2.

**Table 2:** Investigation hypothesis.

Hypothesis	Types of expert		Gender	
	Non-expert	Expert	Women	Men
H: DO^∗^NI^∗^H → PI	Accepted	Accepted	Accepted	Accepted


## Conclusion

The main objective of this research was to determine the influence of the content of the label on the purchase intention of wine. Carrero et al. (2016) highlighted the importance of labels to influence the purchase decision, recommending that companies implement campaigns to inform and educate the consumer about labels’ design and content. The search for strategies that encourage consumer confidence in a product is a key factor for influencing the purchase decision of any given consumer. This research confirmed that the available information on the label can be highly positive in suggesting the purchase intention for different consumers: experts, non-experts, women, and man (Van Den Bulcke and Verbeke, 2001; Hoek and Dewhirst, 2012; Bradu et al., 2014).

This study determined that the information on the label is highly relevant to different types of consumers and to both genders. It is important to emphasize that nutritional information is relevant to differentiate between non-expert and expert consumers, as well as to show differences by gender. Therefore, the nutritional information alone, compared to being combined with other parts of the label, positively influences the purchase intention to a greater extent in experts vs. non-experts and in women vs. men.

In the case of expert consumers, considering all aspects of the label can create a more informed consumer and thus a more informed decision; it is typical that this type of consumer always uses the combined information to better analyze the product (Perrouty et al., 2006). Thus the expert consumer appreciates the existence of detailed information on the label in order to classify the different types of wine into categories (Solomon, 1997).

In the case of the non-expert consumer, though the content of the label can be confusing by not identifying the differences between the wines (and thus leading to the consumer’s failure to classify them by type), it can be concluded that this consumer does not observe the label in order to increase the purchase intention. Still, it is the combination of the denomination of origin and health warnings that together allow the consumer to have some signal to make a decision.

According to Atkin et al. (2007) women are prone to reading labels because they are more guided by the sense of vision. This reading does not necessarily lead to understanding in an intellectual sense, but it makes them feel that they are better informed after considering the label contents (Atkin and Sutanonpaiboon, 2007). Also, as women make decisions influenced by the opinions of others, they possess additional information prior to the time of purchase and, therefore, count on the information from the label to help them identify the characteristics they were seeking (Venkatesh and Morris, 2000). For that reason, in this study it is emphasized that women, especially, rely on nutritional information for their decision making. Women use this information individually or in combination with the other parts of the label such as the denomination of origin, health warnings, and in general combining all parts of the label, whereas men are less visual than women if they actually read the labels.

With respect to the health advisories and in combination with the denomination of origin, it is possible to state that the combined label information is also relevant for increasing the purchase intention, that is, access to more complete information allows for an intuitive selection process based on signals that the consumer can detect (Hass, 1979; Joo et al., 2018).

We have to recognize that our study focuses on the analysis of a small part of the information media that customers can use when making the purchasing decision in the field of wines. We have focused on the label and we have leaved out the analysis other sources of information that can be more recurrent and habitual nowadays for wine experts (digital forums of ethnologists, information from professional journals, etc.). Furthermore, these other sources could contribute more effectively to the purchase decision making of wine. We recognize that the consideration of this media represents a very interesting research line, so future research should address how wine producers could combine different sources of information to influence in the purchase decision.

Other limitation is that this research the study leaves out the application of research techniques related to neuroscience to validate or falsify if the activation of other senses such as smell or taste at the point of sale could be more relevant at the time of produce the experience of wine consumption, and therefore better predict the purchase intention. Therefore, we also propose this possibility for future research because it could complement our results and combining results it will be more accurate a prediction of the purchase intention.

This article presents some recommendations given that the research is important for academics as an aid for the creation and understanding of knowledge about consumer behavior for certain products and markets. Through the disclosure of relevant attributes in the purchase process, these can serve as guidelines to identify patterns in the consumption behavior for other products with characteristics similar to wine.

For future researchers, it is recommended to integrate this research with new discoveries and conclusions related to consumer behavior, through the deepening of the cultural background that allows the identification of differences by country, culture, religion, or race in the consumption of alcoholic beverages. Furthermore, it is proposed as a future line of research to continue examining why in some products the health warnings generate greater consumption, as in the case of wine, that is to say that it does not discourage consumption but rather increases the purchase intention of this product. This aspect can be compared with other alcoholic products to learn if the effect is the same.

For the category of wine, this study provides relevant information about the content that a label must have. Thus, wine producers must pay special attention to the information provided above in order to generate trust and brand awareness in the market. The label content and the reliability of the data must be all-encompassing in order to increase purchase intention, which later can be transformed into successful purchase and profit generation for the producers.

An important bet for producers is to encourage wine tourism, dedicated to advertising the activities related to wine and its production process. This is the perfect opportunity for entrepreneurs to gain more expert consumers and to teach them how to distinguish the attributes of good wine.

Other future paths of research can be associated with the informational accuracy and ethical behavior of wine producers, given the dilemma presented by communicating the real health warnings or nutritional information to consumers and increasing their sales. The accountability for the label information can be explored and the ethical behavior of the wine producer can be considered in this process.

Other future research line is analyze how important is the information of the content of the label in the process of decision to purchase this type of product and compare with other products and it will archive complete this research and make a contribution in this field.

## Author Contributions

DE-B contributed to theory. JR-C contributed to methodology and results.

## Conflict of Interest Statement

The authors declare that the research was conducted in the absence of any commercial or financial relationships that could be construed as a potential conflict of interest.
